# Unifying biology of neurodegeneration in lysosomal storage diseases

**DOI:** 10.1002/jimd.12833

**Published:** 2025-01-17

**Authors:** Anna M. Ludlaim, Simon N. Waddington, Tristan R. McKay

**Affiliations:** ^1^ Department of Life Sciences Manchester Metropolitan University Manchester UK; ^2^ Gene Transfer Technology Group, EGA‐Institute for Women's Health University College London London UK; ^3^ Faculty of Health Sciences Wits/SAMRC Antiviral Gene Therapy Research Unit Johannesburg South Africa

**Keywords:** Fabry disease, Gaucher disease, Krabbe disease, lysosomal storage disease, metachromatic leukodystrophy, mucopolysaccharidosis, neuronal ceroid lipofuscinosis, Nieman‐Pick, Sandhoff, Tay‐Sachs

## Abstract

There are currently at least 70 characterised lysosomal storage diseases (LSD) resultant from inherited single‐gene defects. Of these, at least 30 present with central nervous system (CNS) neurodegeneration and overlapping aetiology. Substrate accumulation and dysfunctional neuronal lysosomes are common denominator, but how variants in 30 different genes converge on this central cellular phenotype is unclear. Equally unresolved is how the accumulation of a diverse spectrum of substrates in the neuronal lysosomes results in remarkably similar neurodegenerative outcomes. Conversely, how is it that many other monogenic LSDs cause only visceral disease? Lysosomal substance accumulation in LSDs with CNS neurodegeneration (nLSD) includes lipofuscinoses, mucopolysaccharidoses, sphingolipidoses and glycoproteinoses. Here, we review the latest discoveries in the fundamental biology of four classes of nLSDs, comparing and contrasting new insights into disease mechanism with emerging evidence of unifying convergence.

## 1 LYSOSOMAL STORAGE DISEASES WITH CENTRAL NERVOUS SYSTEM NEURODEGENERATION

Lysosomal storage diseases (LSD) are a family of inherited rare diseases characterised by the aberrant accumulation of substance in the lysosomes of affected cells. Disease is the result of inherited variants in genes encoding proteins that most often functionally map to vesicular transport or the lysosome. Clinically, LSDs present with multiple morbidities, affecting visceral organs such as the liver and spleen, but with a substantial number also presenting with central nervous system (CNS) neuropathology. CNS neuropathology in LSDs (nLSD) includes impaired axonal transport, synaptic dysfunction and ultimately neurodegeneration. Secondary neuroinflammation has a significant and complex role in many neurodegenerative disorders, including the common neurodegenerative diseases (often historically referred to as ‘age‐related dementias’) and the nLSDs. Astrocytes and microglia function as the immune cells of the CNS, also performing a supportive role for neurons. Reactive gliosis is an inflammatory response involving astrocytes and microglia that aims to limit damage to the CNS but, under conditions of chronic inflammation, can drive a positive feedback loop, amplifying neurodegeneration. In this review, we will focus on intracellular neuronal mechanisms driving LSDs rather than the secondary effects linked to neuroinflammation, which is reviewed elsewhere.^1^ We will concentrate on CNS neurodegeneration, although we recognise that CNS degenerative diseases may exhibit clasmatodendrosis.^2^

Within the nLSDs reviewed here, disease sub‐groups, classified by the substance that accumulates in neuronal lysosomes (Table 1). Possibly the most complex lysosomal aggregate is lipofuscin in the neuronal ceroid lipofuscinoses (NCLs). Lipofuscin content varies and remains incompletely characterised. It is known to be enriched in mitochondrial proteins, notably the subunit c of mitochondrial ATP synthase, phospholipids, glycolipids and cholesterol as well as metal ions such as iron, copper and zinc. Lysosomal lipofuscin is not restricted to the NCLs, it has also been characterised in many common neurodegenerative diseases including CNS neurons in Alzheimer disease and Parkinson disease, as well as photoreceptor cells in patients with age‐related macular degeneration. This suggests lipofuscin accumulation is a shared consequence of convergent biological processes, either inherited or acquired over time.

**TABLE 1:** Lysosomal storage diseases with central nervous system neurodegeneration discussed herein, grouped according to the substance accumulating in the lysosome.

Lysosomal storage disease	Gene	Lysosomal storage material
Neuronal ceroid lipofuscinoses (NCL)		
Congenital	*CLN10*	Lipofuscin
Infantile NCL	*CLN1*, *14*	Lipofuscin
Late infantile NCL	*CLN2*	Lipofuscin
Variant late infantile NCL	*CLN5*, *6*, *7* and *8*	Lipofuscin
Juvenile NCL	*CLN3*, *9*, *12*	Lipofuscin
Adult NCL (Parry, Kufs)	*CLN4*, *11*, *13*	Lipofuscin
Mucopolysaccharidoses (MPS)		
MPS I (Hurler)	*IDUA*	Heparan sulphate
MPS II (Hunter)	*IDS*	Heparan sulphate
MPS IIIA	*SGSH*	Heparan sulphate
MPS IIIB	*NAGLU*	Heparan sulphate
MPS IIIC	*HGSNAT*	Heparan sulphate
MPS IIID	*GNS*	Heparan sulphate
MPS VII	*GUSB*	Heparan sulphate
Gaucher disease (type II and III)	*GBA1*	Glucosylceramide and glucosylsphingosine
Niemann‐Pick type C disease	*NPC1/NPC2*	Cholesterol/glycosphingolipid

Mucopolysaccharidoses (MPS) I, II, III and VII are nLSDs that accumulate glycosaminoglycan substrates, notably heparan sulphate, at the lysosome due to genetic defects in enzymes involved in glycosaminoglycan degradation. Similarly, nLSD sphingolipidoses, including Gaucher types II and III, Niemann‐Pick (types A and C) diseases, are all the result of defects in lysosomal sphingolipid degradation.

nLSDs all show progressive CNS neurological decline with divergent age of onset from infancy to adulthood. CNS neurodegeneration is identified through a plethora of clinical neurological criteria benchmarked against natural history studies. This review focuses on the progress made over recent years in unravelling the molecular mechanisms underlying CNS neurodegeneration in NCL, MPS, Niemann‐Pick type C (NPC) and Gaucher disease (GD). There are many other clinically well‐defined nLSDs equally warranting discussion but with little or no published cell and molecular research. Therefore, we will explore commonalities in cellular dysfunction due to variants in functionally diverse genes causing NCL, MPS, NPC and GD.

### 1.1 Neuronal ceroid lipofuscinosis

The NCLs are a complex group of somewhat overlapping diseases resulting from autosomal recessive inheritance of biallelic variants in 14 genes annotated as *CLN*s. Disease‐causing variants in *CLN9*, *12*, *13* and *14* are rare, clinically complex and overlap with other disease classifications. With a collective incidence of 1:25 000 births^3^ worldwide, they are often considered the most common LSD. Patients with NCLs exhibit CNS neurodegeneration with blindness, seizures, motor and cognitive decline and premature death. Clinically, NCLs are diagnosed and categorised by age of onset into congenital (CLN10), infantile (CLN1), late infantile (CLN2), variant late infantile (CLN5, 6, 7 and 8), juvenile (CLN3) and adult (CLN4 and 11) forms.^3^

The CLN1/PPT1, CLN2/TPP1, CLN5 and CLN10/CTSD proteins are all soluble lysosomal enzymes involved in substrate clearance. Loss‐of‐function gene variants result directly in the characteristic accumulation of storage material seen in LSDs. CLN3, 6, 7 and 8 are transmembrane proteins that all localise to the vesicular transport network.^3^ CLN6 and CLN8 proteins physically interact and collaborate in coat protein complex vesicles that are involved in the transport of lysosomal enzymes, including CLN5, from the ER to the Golgi.^4^, ^5^ CLN3 has also been shown to have a function in vesicular trafficking of lysosomal proteins, including the mannose‐6‐phosphate receptor (M6PR), from the Golgi to the lysosome.^6^ CLN3 and CLN7 proteins share structural homology and are both classified in the Major Facilitator Superfamily of proteins, which include channels and solute transporters. CLN3 has recently been implicated in glycerophosphoinositol catabolism^7^, ^8^ and CLN7 has outward‐facing Cl^−^ channel activity at endolysosomal vesicle membranes.^9^ *CLN11/GRN* encodes progranulin a lysosomal precursor glycoprotein, proteolytically cleaved into seven bioactive granulin peptides (6 kDa) that play an undefined role in the regulation of lysosomal acidification and proteolysis.^10^, ^11^

### 1.2 Gaucher disease

GD has a collective incidence of 1:57 000 births worldwide, but a much higher incidence in the Ashkenazi Jewish population.^12^ Although GD manifests as a spectrum, it has been historically classified into three forms. More than 85% of GD patients are classified as GD1; these patients do not present with neurological features but do commonly present with hepatosplenomegaly, thrombocytopenia, anaemia and osteopenia. Individuals with GD1 often have a normal life expectancy. GD2 and GD3 are infantile‐onset neuronopathic (nGD) disease types featuring acute cortical neurodegeneration. In the case of GD2, this leads to mortality usually before 3 years and GD3 patients die in the third or fourth decade, but with some exceptional patients surviving up until their sixth decade.^13^, ^14^

GD is caused by inheritance of biallelic variants in the GBA1 gene that encodes the lysosomal glucocerebrosidase enzyme. It is distinct from the GBA2 gene, which encodes a non‐lysosomal glucocerebrosidase.^15^ The lysosomal enzyme catabolises glycosphingolipid glucosylceramide (GlcCer) to glucose and ceramide. Many *GBA1* variants are believed to result in defective trafficking of glucocerebrosidase to the lysosome rather than reduced catalytic function at the lysosome.^16^ *GBA1* variants can cause protein misfolding that promotes ER retention and ER‐associated degradation, resulting in the loss of glucocerebrosidase function, lysosomal GlcCer accumulation and neurodegeneration in nGD.^17^, ^18^

GBA1 genotype to phenotype correlation is complex and unresolved. Biallelic GBA1 p.L444P variants can result in all three types of GD^19^, ^20^ as well as monoallelic p.L444P associated with Parkinson disease.^21^ Furthermore, GD1 patients have an increased probability of developing Parkinson disease later in life.^22^ This suggests other factors, including gene modifiers, contribute to disease progression and severity.

### 1.3 Niemann‐Pick disease

Niemann‐Pick disease is an autosomal recessive disease clinically classified into types A, B and C with a shared cellular phenotype of lysosomal cholesterol and glycosphingolipid accumulation in affected cells. Niemann‐Pick A (NPA) and Niemann‐Pick B (NPB) are both caused by variants in the SMPD1 gene, encoding acid sphingomyelinase. This enzyme is responsible for the breakdown of sphingomyelin into ceramide and phosphorylcholine in the lysosome. Loss of function of this enzyme leads to the lysosomal accumulation of sphingomyelin. Individuals with NPB have residual sphingomyelinase activity and, as a result, tend to only present with visceral disease, including hepatosplenomegaly, thrombocytopenia, osteopenia and respiratory issues. Individuals with NPA often have little or no sphingomyelinase activity, and as a result, their presentation is infantile onset and much more severe, including rapid progressive neurodegeneration.

Niemann‐Pick type C disease is caused by loss‐of‐function variants in *NPC1*, *NPC2* genes, which encode proteins that transport low density lipoprotein‐derived cholesterol out of the lysosome.^23^ NPC2 protein binds NPC1 at its second luminal loop and presents lysosomal cholesterol to the N‐terminus of NPC1 under acidic conditions.^24^, ^25^ Variants in *NPC1* and *NPC2* result in the accumulation of lysosomal cholesterol and glycosphingolipids. Both lipids are essential for the maintenance and function of cellular membranes and membrane‐bound organelles, resulting in neuronal destabilisation and subsequent neurodegeneration.

### 1.4 Mucopolysaccharidoses

MPS are a group of autosomal recessive inherited monogenic diseases that cause deficiencies of lysosomal enzymes that catabolise glycosaminoglycans such as heparan sulphate. There are seven types (MPS VII) with varying clinical features but usually including coarse facial features, cognitive retardation and hepatosplenomegaly.^26^ Cortical neurodegeneration is associated with MPS I, II and most substantially III (which is sub‐categorised into MPS IIIA–D), where cortical neurons have enlarged endolysosomal vesicles with glycosaminoglycan accumulation and reduced lysosomal activity.^27^, ^28^

MPS I, II, IIIA, IIIB, IIIC and IIID are the result of variants in IDUA (alpha‐L‐iduronidase), the X‐linked *IDS* (iduronate 2‐sulphatase), *SGSH* (*N*‐sulfoglucosamine sulfohydrolase), *NAGLU* (alpha‐*N*‐acetylglucosaminidase), *HGSNAT* (heparan‐glucosaminide *N*‐acetyltransferase) and *GNS* (glucosamine(*N*‐acetyl)‐6‐sulfatase) genes, respectively. Glycosaminoglycan accumulation results in lysosomal dysfunction, leading to neurodegeneration.

Collectively, these nLSDs are caused by variants in genes encoding proteins with casual or direct links to lysosomal function as well as often substantial clinical overlaps. The individualistic approaches of targeted enzyme replacement therapy and substrate reduction therapy have been highly effective in treating visceral disease in some LSDs, but generally these therapeutics do not cross the blood–brain‐barrier and so cannot be systemically delivered to treat CNS symptoms in LSDs. Gene therapy, where adeno‐associated virus (AAV) vectors expressing the cDNA of the affected gene can be administered intracranially or intrathecally has shown great promise in pre‐clinical models.^29^ However, this has often not translated into clinical trials.^30^, ^31^ This may be another indicator that the successful treatment of nLSDs requires a more nuanced approach, and understanding the linked mechanistic biology could be a route to more efficacious, and potentially unifying therapies. Here, we look at the lysosome and beyond for shared molecular features of nLSDs.

## 2 LINKS BETWEEN nLSDs AND COMMON NEURODEGENERATIVE DISEASES

**FIGURE 1:**
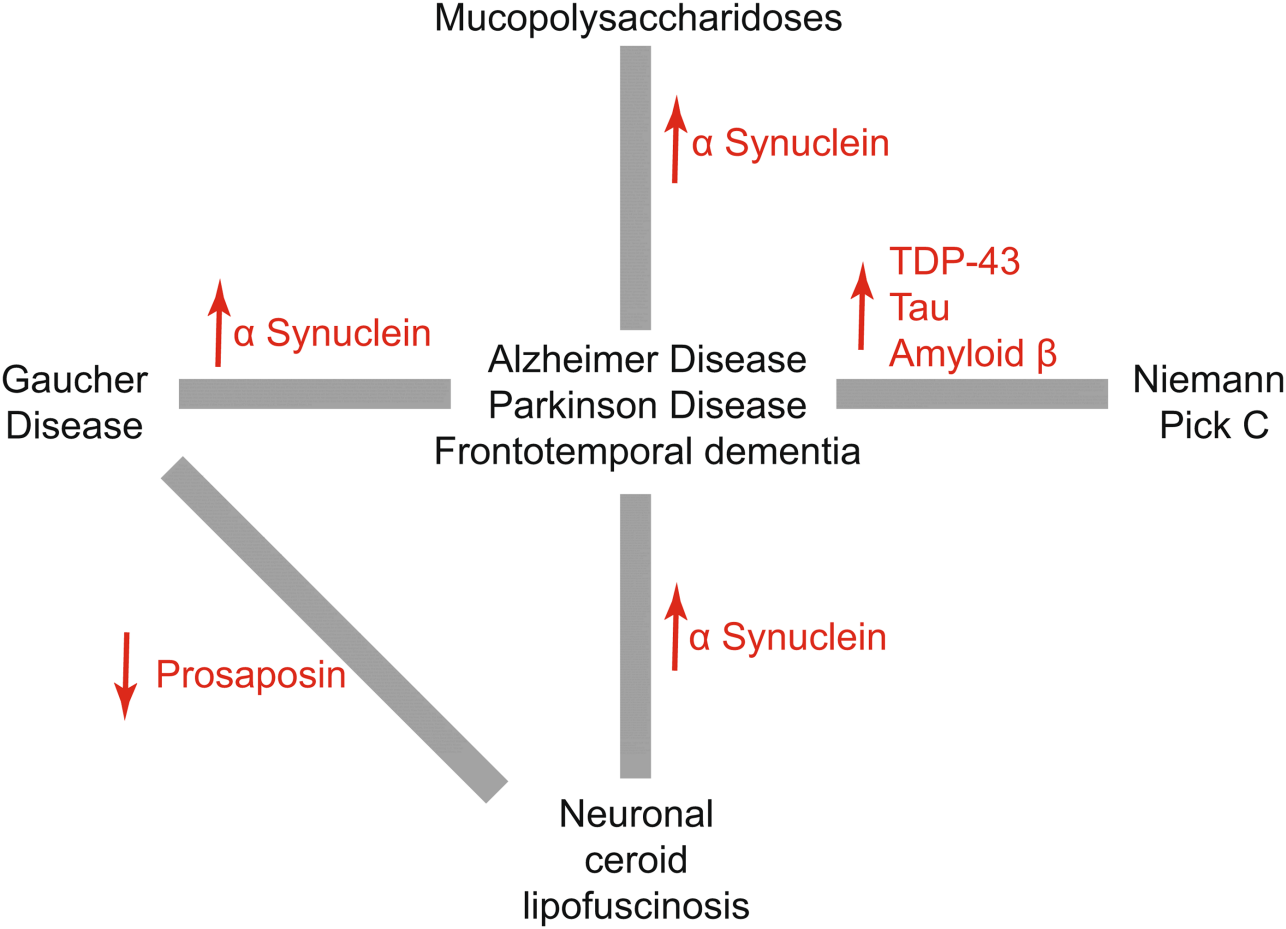
Common substrate accumulations that link lysosomal storage diseases with central nervous system neurodegeneration with common neurodegenerative diseases.

Pathogenic commonalities between neuronal and extracellular accumulation of aberrant protein aggregates in the brains of those affected by synucleinopathies and tauopathies are well known. ⍺‐synuclein aggregates occur in Parkinson disease, amyloid‐β (Aβ42) and hyperphosphorylated tau in Alzheimer disease, and mislocalisation of nuclear transactive response DNA‐binding protein 43kDa (TDP‐43) in frontotemporal dementia and amyotrophic lateral sclerosis (Figure 1).

The link between *GBA1* variants and Parkinson disease risk is well established. The risk that GD patients will develop Parkinson disease is increased compared to the general population^32^ and heterozygous *GBA1* variants are the single largest genetic risk factor for developing Parkinson disease.^21^ Despite the increased disease risk posed by *GBA1* variants, not all GD patients will go on to develop clinical features of Parkinson disease. However, a study by Beavan et al. found that many GD patients exhibit prodromal signs of Parkinson, including parkinsonian motor signs, mild cognitive impairment and olfactory abnormalities.^33^

Degeneration of dopaminergic neurons occurs in the *substantia nigra* of Parkinson disease brains, concomitant with ⍺‐synuclein aggregation and accumulation. The same is observed in rat cortical neurons with *Gba1* depletion.^34^ Deletion of the *GBA1* homologue dGBA1b in Drosophila results in the accumulation of protein aggregates in a ⍺‐synuclein‐independent manner suggesting other factors may play a role in neurodegeneration.^35^ More specifically, *GBA1* variants in Parkinson disease dopaminergic neurons manifest as hyperactivation of mechanistic target of rapamycin complex (mTORC1), a negative regulator of the lysosome‐autophagy network. This results in an autophagic block and elevated ⍺‐synuclein accumulation that is ameliorated by inhibition of acid ceramidase, which deacylates glucosylceramide to glucosylsphingosine (GlcSph), mechanistically linking Parkinson disease and GD.^36^

Interestingly, other LSD genes have been implicated as Parkinson disease risk factors. The study by Robak et al. found that 56% of their Parkinson disease patient cohort had at least one damaging variant in an LSD‐associated gene.^37^ These links are being increasingly recognised and these observations imply shared aetiology between Parkinson disease and nLSDs, with significant overlap in the pathological mechanisms involved in both. The accumulation of ⍺‐synuclein could be cause or effect. *MpsIIIa* knockout mice accumulate insoluble ⍺‐synuclein at the synapses of cortical neurons, the consequence of defective synaptic vesicle recycling through interactions with soluble N‐ethylmaleimide sensitive factor attachment protein receptor (SNARE) family proteins.^38^ This phenotype is partially ameliorated by ablating one *Snca* allele in *MpsIIIa* knockouts.^39^ Observations of inclusions containing ubiquitin and ⍺‐synuclein in post‐mortem brain tissue of MPS III patients are also common.^21^, ^32^ SNARE‐mediated synaptic vesicle fusion and recycling is a neuron‐specific process but is also involved in endolysosomal vesicular trafficking more generally. The relevance of this in nLSDs will be discussed later in greater detail.

As previously mentioned, accumulation of autofluorescent lipofuscin in enlarged lysosomes is a shared characteristic between Parkinson disease and the NCLs suggesting shared molecular pathophysiology. ⍺‐synuclein accumulates in the lysosomes of fibroblasts from both CLN5^40^ and CLN10 patients, where ⍺‐synuclein is a direct degradation target of CLN10. Application of recombinant CLN10/CTSD (Cathepsin D) ameliorates ⍺‐synuclein aggregation and Parkinson disease pathology in ⍺‐synuclein A53T variant‐containing induced Pluripotent Stem Cells (iPSC)‐derived neurons.^41^

CLN7 pathogenic gene variants span a neurological disease spectrum where biallelic missense and nonsense variants result in late infantile NCL and some rare incidences of isolated juvenile macular dystrophy,^42^ whereas haploinsufficiency can result in adult‐onset retinopathies,^43^ amyotrophic lateral sclerosis^44^ and frontotemporal dementia.^45^ A similar situation occurs with *CLN11* (progranulin), where biallelic variants cause the adult‐onset NCL (also known as Kufs disease), but haploinsufficiency results in frontotemporal dementia. CLN11 is a complex multifunctional protein with intracellular chaperone activity, including the transport of the saposin precursor prosaposin to the lysosome, where it acts as a glucocerebrosidase co‐factor in the enzymatic degradation of glycosphingolipids.^46^ This is the lysosomal degradative pathway that is disrupted in GD, but reduced levels of prosaposin are also observed in frontotemporal dementia patients.^46^ Indeed, frontotemporal dementia patients with *CLN11* haploinsufficiency, and *Pgrn* ^−/−^ mice, have decreased lysosomal glucocerebrosidase activity, which can be rescued by ectopic CLN11 expression.^47^ CLN5 genetic variants are an established Alzheimer disease risk factor.^48^ CLN5 loss of function results in reduced processing and activity of lysosomal CLN10, which may target and degrade Aβ42, preventing its pathological accumulation.^48^, ^49^

Niemann‐Pick C shows some similarities in molecular pathology with Alzheimer disease. Aβ42 accumulation, tau pathology and TDP‐43 mislocalisation are all also observed in NPC patient neurons.^50^, ^51^ Depletion of NPC1 massively enhances the transport of tau across the plasma membrane of neurons, potentiating prion‐like tau transfer.^52^ Kagedal et al. report that NPC1 is upregulated in the brains of Alzheimer disease patients and hypothesise that this is a compensatory effect due to altered cholesterol homeostasis.^53^ There also exist links between NPA/NPB and Alzheimer disease. Lee et al. report that the activity of acid sphingomyelinase, encoded by SMPD1, is increased in Alzheimer disease patients and that partial inhibition reduces the pathology seen in Alzheimer disease mice.^54^

There are further relatively unexplored connections between Parkinson clinical criteria and nLSDs. In fact, adult‐onset nLSDs including Niemann‐Pick can be misdiagnosed as atypical parkinsonism.^55^ Furthermore, LSD patients often have movement disorders similar in presentation to Parkinson disease that doubtless relate to peripheral neuronal degeneration but remain insufficiently investigated.^56^

## 3 COMMON DEFECTS IN NEURONAL CELL FUNCTION IN nLSDs

**FIGURE 2:**
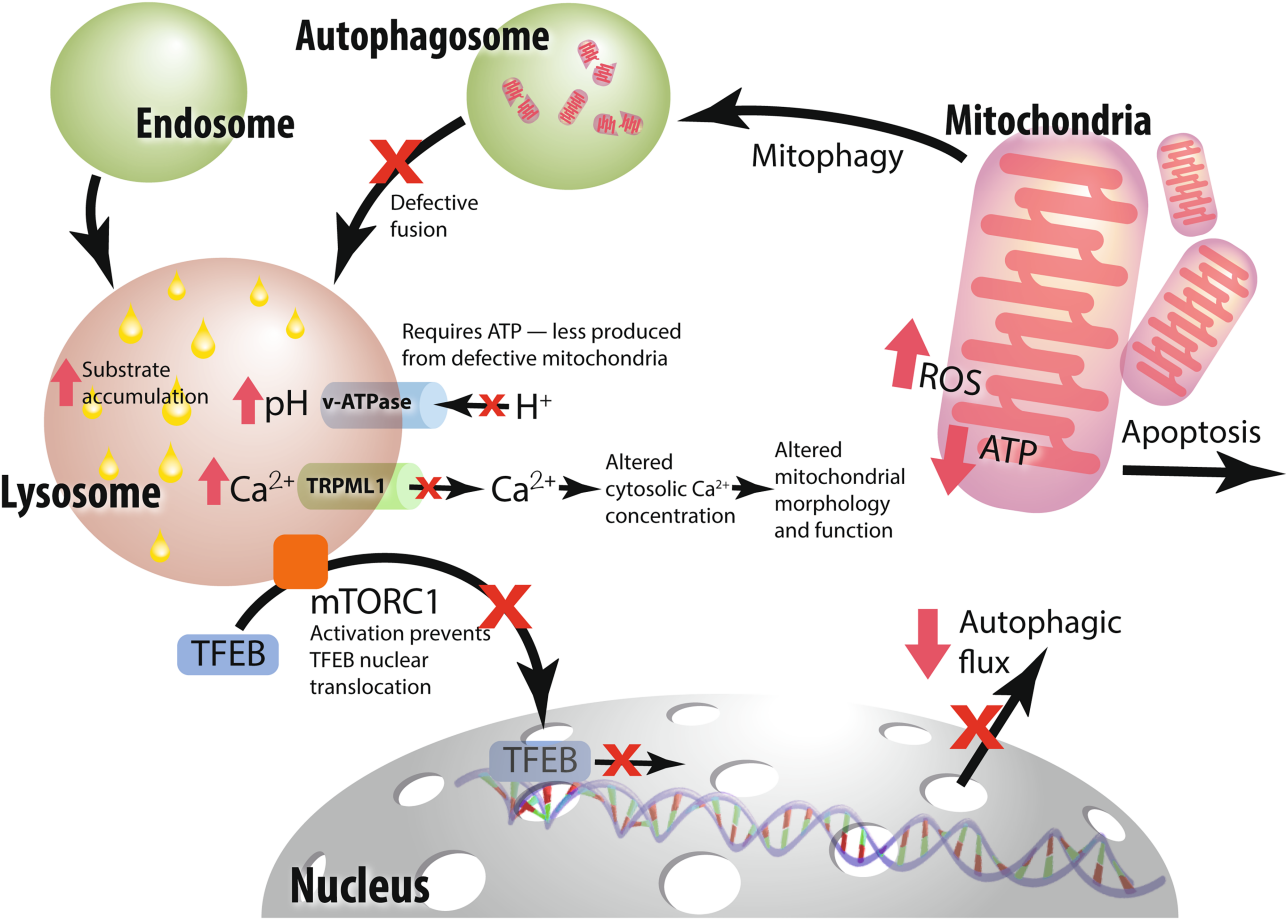
Intracellular networks common to the lysosomal storage diseases with central nervous system neurodegeneration (nLSDs). Lysosomal dysfunction results in downstream effects that result in feedback loops impacting vesicular transport and autophagy. nLSDs are associated with mechanistic target of rapamycin complex (mTORC1) hyperactivation, inhibiting transcription factor EB box (TFEB) nuclear translocation, in turn preventing the transcription of genes involved in autophagic flux. Substrate accumulation within the lysosome results in alkalinisation of the lysosomal lumen, meaning the loss of the optimal pH for lysosomal enzyme function. Further lysosomal alkalinisation occurs as reduced ATP production from defective mitochondria means reduced activity of the ATP‐dependent v‐ATPase proton pump that acts to maintain lysosomal pH. Additionally, calcium efflux from the lysosome into the cytosol is reduced. Disruption of calcium homeostasis results in alterations to mitochondrial morphology and function, triggering mitophagy. Defective mitochondria are enveloped in the autophagosome but are only partially degraded, preventing effective organellar recycling. Autophagosome‐lysosome fusion defects result in the accumulation of defective autophagosomes.

Lysosomal dysfunction, through the accumulation of storage materials, results in secondary effects on vesicular transport, autophagy, cell signalling and mitochondrial function (Figure 2). Lysosomal defects are considered the central cause of neurodegeneration in nLSDs and sometimes the connection between gene variant and lysosomal defects is direct. For example, NPC1 and 2 proteins are involved in cholesterol export from the lysosomal lumen, and pathogenic variants cause the aberrant accumulation of lysosomal cholesterol and sphingolipids.

The nGD (*GBA1*), nMPS (*IDUA*, *IDS*, *SGSH*, *NAGLU*, *HGSNAT*, *GNS* and *GUSB*) and NCL (*CLN1*, *CLN2*, *CLN5* and *CLN10*) genes all encode lysosomal enzymes. Pathogenic loss of function results in substrate accumulation and lysosomal dysfunction. Other NCLs are more complex. CLN3 and CLN7 proteins have been implicated in lysosomal functions, but how loss of function leads to neurodegeneration is unclear. CLN6 and CLN8 proteins are more indirectly linked via the vesicular transport of lysosomal proteases and CLN11 in modulating vacuolar‐type ATPase activity and hence lysosomal acidification.^10^ This new research provides good evidence that defects in lysosomal protein trafficking can result in lysosomal defects and neurodegenerative substance accumulation. Conversely, there is equally compelling evidence that lysosomal dysfunction results in negative feedback loops to endosomal vesicular transport and autophagy networks.

## 4 ENDOLYSOSOMAL VESICLE TRANSPORT AND FUNCTION

Lysosomal proteins are transported from the ER to the trans‐Golgi vesicle network for post‐translational modification and to the lysosomal compartment via endosomal vesicular transport. Some nLSD variants result in ER‐mediated quality control mechanisms labelling misfolded protein for degradation. NPC1 is normally stabilised by Hsp70 and Hsp90 chaperone proteins, but pathogenic variants lead to elevated Hsp70 binding and proteasomal degradation.^57^, ^58^ Similarly, in nGD, mutated misfolded glucocerebrosidase binds to Hsp27 in preference to LIMP2 association, which directs its proteasomal degradation.^59^, ^60^ In MPS II, *IDS* variants are trapped in the ER and directed to ER‐associated degradation.^61^

Proteins produced at ribosomes on the ER are transported to the Golgi by coat protein complex II (COP II) vesicles for post‐translational modifications. As previously stated in the NCLs, CLN6 is an ER‐associated protein that directly interacts with CLN8 to form complexes with lysosomal enzymes, including CLN5, to facilitate COP vesicle‐mediated ER to Golgi transport.^4^, ^5^ CLN1 catalyses depalmitoylation of s‐palmitoylated proteins, which can form part of the process of proteasomal degradation at the lysosome. However, Bagh et al. recently showed that two key components of the regulator mTORC1 nutrient‐sensing complex; vacuolar‐type ATPase and Lamtor1 are misrouted to the plasma membrane rather than lysosomal membrane by aberrant endosomal trafficking in *Cln1* ^−/−^ cells.^62^ Interrogation of CLN3 biology has been hampered by the lack of effective endogenous antibodies but there is evidence of its involvement in endosomal transport through interaction with the late endosome Rab‐GTPase RAB7A.^63^ Furthermore, Calcagni et al. describe CLN3 as a lysosomal sorting receptor that interacts with the cation‐independent mannose‐6‐phosphate receptor (CI‐M6PR) to transport it and its lysosomal enzyme cargoes from Golgi to lysosome.^6^

NPC1 also interacts with RAB7A to mediate cholesterol export from the lysosome^64^ where altered cholesterol levels destabilise the membrane distribution of RAB7A, affecting endosome‐lysosome fusion.^65^ In NPC1 patient fibroblasts, sphingosine accumulates in late endosomes and lysosomes, reducing TRPML1‐mediated Ca^2+^ release and impeding transcription factor EB box (TFEB)‐mediated lysosomal biogenesis.^66^, ^67^, ^68^ Lysosomal Ca^2+^ export via TRPML1 is also reported as a result of CLN7 acting as an outward‐facing chloride channel in endolysosomal vesicle membranes, where its influence on lysosomal acidification induces TRPML1.^9^

Endosomal and synaptic vesicle biology significantly overlaps, and it is logical that pathogenic variants affecting one also affect the other. The formation of membrane‐encapsulated vesicles within the endosomal and autophagic networks, as well as synaptic vesicles is initiated by the formation of endosomal sorting complex required for transport (ESCRT) complexes at intracellular membranes. CLN3 is implicated in the sorting and transport of the Kv4 potassium channel at neuronal synapses^69^ and Cln7 knockout in Drosophila results in defective synaptic vesicles.^70^ This could represent further evidence for neuronal susceptibility in NCL and cross‐connectivity where gene defects affect both endosomal and synaptic vesicle function. CLN4 is proposed to be a chaperone protein in synaptic vesicles and accumulates at endolysosomal vesicles in CLN4 patient cells.^71^ CLN4 promotes ESCRT complex‐mediated sorting of synaptic proteins and CLN4 disease‐causing variants in Drosophila lead to neurodegeneration as a result of the accumulation of lipofuscin‐containing misfolded CLN4 protein.^72^

The fusion of vesicles in the endolysosomal network is critical for cellular homeostasis and controlled primarily by two key protein complexes. In concert with Rab‐GTPases and SNARE complex proteins, the class c core endosomal vacuole tethering (CORVET) complex enables early to late endosome fusion and interaction with the homotypic fusion and vacuole protein sorting (HOPS) complex to enable vesicular fusion of both endosomes and autophagasomes with the lysosome.^73^ CORVET and HOPS share core proteins including vacuolar protein sorting (VPS)‐associated proteins, and facilitate vesicular biogenesis, transport, fusion and recycling. VPS33A is one of the central core components of CORVET/HOPS, and pathogenic variants result in MPS‐like disease with elevated lysosomal heparan sulphate^74^ with reduced endocytic transport of ceramide in affected neurons.^75^ VPS16 variants cause MPS‐like disease with endolysosomal defects in neurons and reduced VPS33A expression.^76^ VPS53 is a component of the Golgi‐associated retrograde protein (GARP) complex involved in protein sorting and glycosylation at the Golgi. Variants in VPS53 result in reduced NPC2 protein, altered cholesterol distribution and block the recycling of CI‐M6PR to the Golgi.^77^ NPC1 is necessary for Rab8a‐mediated segregation of late endosomes from lysosomes,^78^ and lysosomal recycling is perturbed in *GBA1*‐mutated Parkinson disease fibroblasts,^79^ showing, in principle, the same could be true in nGD.

Lysosomal neurodegenerative phenotypes shared between patients with variants in VPS16, VPS33A, VPS53, NCL, MPS and NPC genes strongly imply a mechanistic convergence on defects in vesicular transport and endolysosomal fusion, significantly contributing to lysosomal dysfunction.

## 5 AUTOPHAGY

Whilst endosomes transport membrane‐bound proteins and enzymes from the ER/trans‐Golgi network to the lysosomal compartment as their functional destination, autophagy is the process of encapsulating unwanted proteins and organelles to the lysosome for breakdown and recycling. Lysosomal dysfunction has the knock‐on effect of autophagic shunting, whereby autophagosome biogenesis continues but the process of autolysosome fusion is prevented. This leads to an accumulation of enlarged autophagosomes unable to dock and fuse with the lysosomal membrane. This is a shared feature of nLSDs where the accumulation of p62^+^ autophagosomes in cortical neurons is synonymous with the concomitant accumulation of LAMP1/2^+^ lysosomes,^80^, ^81^ leading to neuronal apoptosis.^82^ *GBA1* is a direct transcriptional activation target of the TFEB, the master regulator of lysosomal biogenesis.^83^ *GBA1* over‐expression leads to elevated autophagic flux and induces apoptosis,^84^ although autophagic shunting is not described. Consistent with clinical outcomes, elevated autophagosome accumulation is observed in iPSC‐derived neurons generated from GD2 and GD3 patients, but not in non‐neuronopathic GD1.^85^ This implies differential cellular mechanisms between neuropathic and non‐neuropathic GD but does not address how genotype/phenotype correlations can be so divergent.

The link between NPC1/2 and lysosomal cholesterol export is well established,^23^ where NPC1/2 variants result in the accumulation of lysosomal cholesterol and glycosphingolipids. Mechanistically, NPC1 binds SLC38A9 at the lysosome membrane and inhibits mTORC1 signalling.^86^ NPC1 depletion leads to constitutive mTORC1 activation, suppressing autophagy.^87^ Additionally, NPC1 deficiency results in impaired autophagosome‐lysosome fusion which has been linked to impaired lysosomal proteolysis.^88^ Others argue that the process is a proteolysis‐independent, but SNARE‐dependent mechanism.^89^

## 6 MITOCHONDRIA

Mitochondrial defects are common in neurodegenerative diseases, primarily because neurons have much higher energy demands than nearly all other cell types, and therefore subtle alterations to mitochondrial functional efficiency can have catastrophic effects. This can either be the result of directly debilitating effects in energy production through oxidative phosphorylation or more indirect effects on the genesis, maintenance and timely degradation of mitochondria.

The nLSDs all present with mitochondrial defects and connectivity between the lysosome and mitochondrial membranes through membrane contact sites (MCS) could be a rational mechanism for lysosomal defects translating to mitochondrial dysfunction. NPC1/2 variants prevent normal cholesterol transport from the lysosome. In mice, *Npc* depletion results in reduced plasma membrane cholesterol content^90^ and reduced endosomal transport of cholesterol to the mitochondria.^91^ Additionally, aberrant transport of lysosomal cholesterol in NPC leads to damaging redistribution of the calcium channel inosiltol 1,4,5‐triphosphate receptor, IP(3)R1, on the membranes of the endoplasmic reticulum. This results in elevated Ca^2+^ release into the cytosol potentially causing mitochondrial dysfunction due to Ca^2+^ toxicity.^92^ Interestingly, IP(3)R1 expression is reduced in Cln1^−/−^ cells, leading to defects in Ca^2+^ transport from the endoplasmic reticulum to the lysosome, although mitochondrial defects are not specifically mentioned.^93^ Contrastingly, Hoglinger et al. report higher concentrations of cholesterol at lysosome‐mitochondrial MCS and elevated cholesterol in mitochondria.^94^ An alternative mechanism is that aberrant cholesterol distribution at endoplasmic reticulum‐lysosome MCS prevents the distribution of voltage‐gated calcium channels, and this causes, or contributes to mitochondrial toxicity.^95^ Whether higher or lower, cholesterol imbalance reduces the functional permeability of mitochondrial membranes, affecting oxidative phosphorylation and elevating apoptosis.^96^

Mitochondrial biogenesis, itself, is negatively affected in NPC patient cells. Elevated KLF2 and ETV1 act as transcriptional repressors of this process^97^ whilst cholesterol accumulation at the lysosome causes hyperactivation of mTORC1 signalling and reduced mitophagy.^98^ The lysosomal accumulation of the subunit c of mitochondrial synthase and elevated reactive oxygen species are neuronal hallmarks in the NCLs and hallmarks of defective mitophagy. Our own work in Cln7^−/−^ cortical neurons and iPSC‐derived NPCs showed that elevated reactive oxygen species stabilises 6‐phosphofructo‐2‐kinase/fructose‐2,6‐biphosphatase 3 (PFKFB). This diverts glucose metabolism to glycolysis rather than through the pentose phosphate pathway. In turn, this promotes an antioxidant response, creating a negative feedback loop.^99^

A similar cellular phenotype is observed in mice harbouring a single allele of the L444P *Gba1* variant, whereby defective mitophagy results in increased mitochondrial reactive oxygen species.^100^ *Gba1* ^−/−^ neurons present reduced mitochondrial Ca^2+^ uniporter expression, have reduced mitochondrial Ca^2+^ uptake, and hence collapse of mitochondrial membrane potential, decreased oxidative phosphorylation and increased production of reactive oxygen species.^101^ More specifically, there is good evidence that glucocerebrosidase is targeted directly to the mitochondria in an Hsp60‐dependent manner where it stabilises mitochondrial respiratory complex I.^102^ The evidence to date implies both direct and indirect roles in mitochondrial biogenesis, function and turnover result in or contribute to nLSD.

## 7 ORGANELLAR LIPID ACCUMULATION

Cholesterol and sphingolipids are critical components of the plasma membrane and intracellular membranes. In the NCLs, *CLN11* variants lead to lysosomal glycosphingolipid accumulation due to defective processing and delivery of saposin, which is essential for lysosomal glycosphingolipid degradation.^46^ Sphingolipid and glycerophospholipid metabolism is modulated in CLN6.^103^ *CLN3* knockout results in decreased activity of lysosomal lipid‐degrading enzymes, resulting in altered distribution of membrane lipids such as lactosylceramides and glycosphingolipids.^104^ Interestingly, in *Cln3* knockout mice, the blood–brain‐barrier becomes permeable due to reduced plasma membrane cholesterol content implying cellular membrane connectivity and cholesterol communication from lysosomal membranes to plasma membrane.^105^ Most recently, a breakthrough in lysosomal isolation and next‐generation proteomic evaluations showed that *CLN3* knockout resulted in the lysosomal accumulation of glycerophosphodiesters, the end products of glycerophospholipid catabolism.^7^ Nayame et al. subsequently showed that glycerophosphodiesters competitively inhibit lysosomal phospholipase‐mediated glycerophospholipid catabolism. Therefore, toxic phospholipids accumulate in the lysosome and may represent the primary cause of disease in juvenile NCL.^8^

The link between lipid dysregulation and MPS is less obvious, but lysosomal lipid accumulation has been observed in MPS IIIB.^106^ In GD, *GBA1* variants result in lysosomal accumulation of GlcCer and GlcSph,^107^ also resulting in altered membrane sphingolipid composition in GD fibroblasts.^108^ Moreover, in GD fibroblasts, GlcCer and GlcSph substrate leak out of the lysosome to the cytosol and act as a substrate for the non‐lysosomal *GBA2*. This results in the cytosolic accumulation of sphingosine, which inhibits GBA2 in a negative feedback loop.^109^

Cholesterol accumulates at the lysosome as a consequence of protease‐driven hydrolysis, and NPC1 and NPC2 are well documented as mediators of cholesterol efflux from the lysosomal lumen and negative regulators of mTORC1.^87^, ^98^ Phenotypically, cholesterol accumulation at lysosomal membranes impedes their axonal transport in NPC neurons.^110^ Cholesterol transporters, including phosphatidylinositol 4‐phosphate (PtdIns4P), are negatively affected by NPC1 variant, which disrupts the recycling of cholesterol along lysosomal‐ER and Golgi‐ER MCS^111^ and reduced plasma membrane cholesterol content, capable of aberrantly activating Src kinase and endocytosis.^90^ The priming and boosting of the Stimulator of interferon genes (STING) protein expression has been shown to occur in NPC. STING is a master regulator of the innate immune response to pathogen infection through the identification of cytosolic double‐stranded DNA, initiating a process leading to apoptosis. NPC1 depletion enables the non‐canonical tethering and trafficking of STING by the sterol regulator element‐binding protein 2 (SREBP2) transcription factor, leading to neuronal apoptosis.^112^ A potentially unifying hypothesis has recently been published by Wang et al. where cells with disease‐causing variants for NCL (*CLN10*), NPC (*NPC1*) and GD (*GBA1*) all presented with a common downstream phenomenon of activating the canonical cGAS‐STING pathway leading to neuronal apoptosis. This cellular mechanism was induced by cytosolic double‐stranded DNA fragments that could be the result of the release of fragmented mitochondrial DNA from leaky, cholesterol‐depleted and lysosomal membranes.^113^

We have discussed commonalities of lysosomal, mitochondrial and cholesterol disease phenotypes across multiple nLSDs, showing how variants in loosely connected genes can converge on a unified mechanistic programme for neurodegeneration. There are now emerging commonalities between NCL, NPC, GD ad MPS, where gene defects result in cellular destabilisation upstream of lysosomal function. Intracellular Ca^2+^ distribution and lipid content of intracellular membranes result in aberrant cell signalling (mTORC1, TFEB), protein transport through MCS and vesicular transport, as well as defective autophagy/mitophagy. Any of these processes could initiate neurodegeneration through apoptosis, individually or collectively. Equally, targeting one or more of these aberrant molecular processes with small molecule or RNA inhibitors could tip the balance and slow neurodegeneration. There lies the potential for similar unification of therapeutic interventions. The Wang study presents a compelling pathogenic connectivity across nLSDs, including NCL, NPC and GD disease models, whereby intrinsic stimulation of the cGAS‐STING innate immune response results in neuronal apoptosis. This exciting discovery could represent a universal target for therapeutic inhibition to extend the lifespan of neurons across all nLSD and exemplifies how an understanding of unifying biology can inform the future intelligent design of the next generation of therapies.

## AUTHOR CONTRIBUTIONS

AML, TRM and SNW each contributed to the drafting of this manuscript.

## CONFLICT OF INTEREST STATEMENT

The authors have no conflict of interest.

## ETHICS STATEMENT

This article does not contain any studies with human or animal subjects performed by any of the authors.
